# Erratum

**DOI:** 10.1155/1994/92038

**Published:** 1994


					ERRATUM
HPB Surgery, 1991, volo 4(2), pp. 137-146.
Paper title: Hepatic Surgery Facilitated by a New Jet Dissector
by H. U. Baer, G. J. Maddern and L. H. Blumgart
The above mentioned publication dealt with the development and successful clinical application of
a novel ultra-high-pressure water-jet-dissector in hepatic surgery. Although the delivering firm
"MEDICAL EXPORTS" was mentioned in a footnote, the text gave the erroneous impression that
the key technical development concerning the water-jet dissector was made by the authors.
Table 1 and Figures 2, 3a and 3b appearing in this manuscript were actually generated by
Mr. A. Neracher, Meditech AG, CH-1247 Anires, Switzerland. Mr. Neracher is the developer of
this apparatus and the owner of the patents.
The authors and the publisher sincerely apologize for this omission.

				

